# Hodgkin Reed–Sternberg-Like Cells in Non-Hodgkin Lymphoma

**DOI:** 10.3390/diagnostics10121019

**Published:** 2020-11-27

**Authors:** Paola Parente, Magda Zanelli, Francesca Sanguedolce, Luca Mastracci, Paolo Graziano

**Affiliations:** 1 Pathology Unit, Fondazione IRCCS Ospedale Casa Sollievo della Sofferenza, 71013 San Giovanni Rotondo, Italy; paolaparente77@gmail.com (P.P.); p.graziano@operapadrepio.it (P.G.); 2 Pathology Unit, Azienda USL-IRCCS Reggio Emilia, 42123 Reggio Emilia, Italy; magda.zanelli@ausl.re.it; 3 Pathology Unit, Azienda Ospedaliera-Universitaria OO.RR, 71100 Foggia, Italy; 4 Anatomic Pathology, Ospedale Policlinico San Martino IRCCS, 16132 Genova, Italy; mastracc@hotmail.com; 5 Anatomic Pathology, Department of Surgical Sciences and Integrated Diagnostics (DISC), University of Genova, 16132 Genova, Italy

**Keywords:** Reed-Sternberg cell, Reed-Sternberg-like cell, classic Hodgkin Lymphoma, CD30, T cell lymphoma, B cell lymphoma, lymphoma diagnosis, lymphoma diagnostics

## Abstract

Reed–Sternberg cells (RSCs) are hallmarks of classic Hodgkin lymphoma (cHL). However, cells with a similar morphology and immunophenotype, so-called Reed–Sternberg-like cells (RSLCs), are occasionally seen in both B cell and T cell non-Hodgkin Lymphomas (NHLs). In NHLs, RSLCs are usually present as scattered elements or in small clusters, and the typical background microenviroment of cHL is usually absent. Nevertheless, in NHLs, the phenotype of RSLCs is very similar to typical RSCs, staining positive for CD30 and EBV, and often for B cell lineage markers, and negative for CD45/LCA. Due to different therapeutic approaches and prognostication, it is mandatory to distinguish between cHL and NHLs. Herein, NHL types in which RSLCs can be detected along with clinicopathological correlation are described. Moreover, the main helpful clues in the differential diagnosis with cHL are summarized.

## 1. Introduction

**Figure 1:**
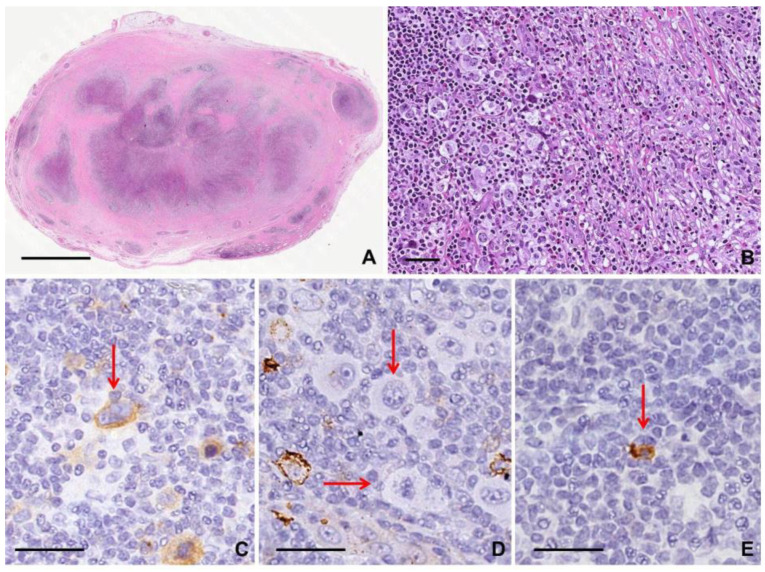
cHL: (**A**) Nodular pattern of growth with sclerosis in nodal cHL (hematoxylin/eosin, 0.5×; scale bar: 0.5 cm). (**B**) RSCs in a typical context of polymorphic infiltrate comprising small lymphocytes, histiocytes, plasma cells, and eosinophils (hematoxylin/eosin, 20×). RSCs (red arrows) with strong expression of CD30 (**C**), negativity for CD20 (**D**), and positivity for EBV/LMP1 (**E**) (immunostaining, 40×; scale bar: 50 µm).

The Reed–Sternberg cell (RSC) is associated with classic Hodgkin lymphoma (cHL) [1]. The typical size of diagnostic RSC is large (15 to 45 micrometers), with abundant slightly basophilic or amphophilic cytoplasm and two or more nuclei. The nuclei are large and often round in contour, with a prominent, often irregular nuclear membrane, pale chromatin and usually one prominent eosinophilic nucleolus, with perinuclear clearing (halo), resembling a viral inclusion [2]. The Hodgkin cell is a mononuclear variant of the RSC; it is characterized by a single round or oblong nucleus with large inclusion-like nucleolus [1]. Some RSCs showing condensed cytoplasm and pyknotic reddish nuclei are known as “mummified cells”. RSCs surrounded by formalin retraction artifact are termed “lacunar cells”. The latter are characteristic of the nodular sclerosing cHL subtype [1,2]. Neoplastic RSCs comprise only 1% of the tumor mass; the majority of the infiltrate is made up of non-neoplastic T cells, B cells, eosinophils, neutrophils, macrophages, and plasma cells. This inflammatory background is vital to the clinical behavior of RSCs as there is bidirectional signaling between cells and environment [1,3]. It is difficult to study the molecular profiling of RSCs for their paucity into the tumor tissue. The major hypothesis, supported by evidence, is that RSC originates from a preapoptotic germinal center B cell with abnormality leading to a “crippled” B cell program expression [4]. This underlying biology contributes to the distinctive RSC immunophenotype CD20− (or focally and weakly positive), PAX5+ (weakly expression compared to B cells), and MUM1/IRF4+, BOB1-(positive in 50% of cases) [1,5]. The typical RSC is CD30+, CD15+, CD45/LCA−, OCT2−, and CD3− (although T cell antibodies may be positive in up to 20% cHL). RSC is mostly positive for Epstein–Barr Virus (EBV) as demonstrated by EBV-encoded small RNA (EBER) or EBV-encoded latent membrane protein-1 (LMP1), with different rates of expression according to different subtypes [1,4,5] (Figure 1).

cHL represents 95% of all Hodgkin lymphomas and has a bimodal age distribution with a peak in young patients where it accounts for 15% of all malignancies, and a second peak in older adults (about 45–50 years) [1,6]. cHL is a nodal disease with virtually all cases arising in peripheral lymph nodes. Other possible primary sites are mediastinum and lung [7]. B-symptoms (fever, weight loss, and night sweats) are frequent at onset. At advanced stage, cHL can involve the spleen, liver, and other extranodal sites, but is always associated with nodal disease [7]. Thus, cases morphologically resembling cHL, but arising in extranodal sites, must be carefully evaluated to exclude B cell or T cell NHLs [6,7]. Bone marrow (BM) involvement is infrequent [7]. Correlation with EBV infection shows contrasting rates, ranging from 90–100% in Latin America, Africa, and Asia, to up to 50% in Western Europe and North America [8]. Doxorubicin, Bleomycin, Vinblastine, and Dacarbazine (ABVD) polychemotherapy and radiotherapy are the standard treatments for cHL [1,7,8] supported by autologous hematopoietic stem cell transplantation in at least 50% of patients [1].

Reed–Sternberg-like cells (RSLCs) are found in many NHLs, such as Anaplastic Large Cell Lymphoma (ALCL) and other T cell lymphomas, as well as in low-grade and high-grade B cell lymphomas [5,7]. As such the differential diagnosis in these cases can be challenging. A correct lymphoma classification is mandatory for adequate therapeutic management [5,7]. The aim of this review is to describe the lymphoproliferative disorders in which RSLCs can be found, according to the current World Health Organization (WHO) classification of tumors of hematopoietic and lymphoid tissues [9]. The main clinical, histological, and immunohistochemical features are presented, providing useful tips in the differential diagnosis between cHL and NHLs featuring RSLCs.

## 2. T Cell Lymphomas

### 2.1. Systemic Anaplastic Lymphoma Kinase-Positive Anaplastic Large Cell Lymphoma (ALK+ ALCL)

#### 2.1.1. Epidemiology and Clinical Features

ALK+ ALCL is a rare subtype of NHL, defined as a distinct type of peripheral T cell lymphoma (PTCL) in the current WHO classification [9]. It mostly affects children and young adults with a male predominance (male/female ratio: 3.0), accounting for 10–15% of pediatric and adolescent NHLs; it represents approximately 3% of adult NHLs [10]. Patients usually present at an advanced stage (stage III or IV), with severe systemic symptoms (75%) [11]. ALK+ ALCL is typically a nodal lymphoma (90% of cases), while extranodal involvement is observed in 60% of cases. The most common extranodal sites include skin (26%), bone (14%), soft tissue (15%), lung (14%), and BM (10–14%) [10,11]. First-line therapy for patients with ALK+ ALCL includes anthracycline-containing CHOP-like regimens or CHOP-like regimens with etoposide [12]. ALK+ ALCL has a better prognosis than other T cell lymphomas, with a 5-year overall survival (OS) of 70–85% [12] (Supplementary Table S1).

#### 2.1.2. Histological Findings and Immunophenotype

**Figure 2:**
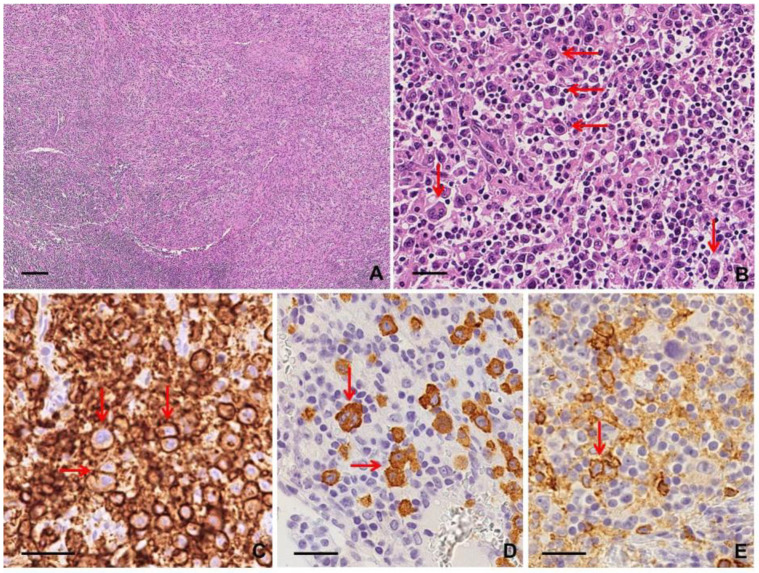
ALK+ ALCL: (**A**) Diffuse pattern of growth in nodal ALK+ ALCL (hematoxylin/eosin, 5×; scale bar: 100 µm). (**B**) RSLCs (red arrows) in a polymorphic infiltrate comprising small lymphocytes and plasma cells (hematoxylin/eosin, 30×; scale bar: 50 µm). RSLCs (red arrows) with strong expression of CD30 (**C**), strong positivity for ALK (**D**), and CD4 (**E**) (immunostaining, 40×; scale bar 50 µm).

Lymph node involvement by ALK+ ALCL is characterized by cohesive clusters of large tumor cells within sinusoids and paracortical area, highlighted by CD30 and ALK immunostains. In extranodal sites, tumor cells show a diffuse growth pattern often with necrosis [9]. A variable number of “hallmark” cells showing abundant cytoplasm and large horseshoe-shaped nuclei with multiple nucleoli are present [13]. By definition, neoplastic large cells are CD30+ with a membrane and dot-like/Golgi pattern. ALK positivity can show a variable staining, depending on the fusion pattern: nuclear and cytoplasmic ALK co-expression correlates with NPM1-ALK fusion, while cytoplasmic staining indicates a gene partner as PABPC1-ALK and EEF1G-ALK [13]. Despite the mature derivation of T cells, neoplastic large cells usually do not express a complete mature T cell phenotype; CD2, CD5, and CD4 are the most frequently expressed T cell markers (40–70%), while CD3 is negative in more than 75% of the cases [9,13]. CD45/LCA is variably positive and EMA expression is common; CD15 is rarely expressed [13]. RSLCs may be present in the common/classic pattern and in the Hodgkin-like pattern. The latter shows an architecture similar to cHL (nodular sclerosis type), with RSLCs embedded within a polymorphous inflammatory background containing eosinophils or neutrophils [12] (Supplementary Table S1; Figure 2).

#### 2.1.3. Clues for Differential Diagnosis with cHL

An important clue is the different growth pattern of neoplastic cells: a sheet-like growth pattern consisting predominantly of large pleomorphic cells with admixed “hallmark” cells in ALK+ ALCL, while scattered RSCs in an appropriate inflammatory background in cHL [9,12]. However, cHL with syncytial growth pattern may mimic the cohesive sheet-like growth of ALCL, and sometimes ALCL may show a nodular/sclerotic growth pattern [5]. By definition, both tumors strongly express CD30, but the expression of PAX5, in addition to CD15 and EBV−LMP1/EBER expression, strongly supports a diagnosis of cHL, while expression of T cell-associated markers or cytotoxic markers supports a diagnosis of ALCL; finally, the staining for ALK protein is decisive for the diagnosis of ALK+ ALCL [9,12]. Moreover, the typical RSC is CD45/LCA− and EMA−, unlike RSLC in ALK+ ALCL [11] (Supplementary Table S1).

### 2.2. Anaplastic Lymphoma Kinase-Negative Anaplastic Large Cell Lymphoma (ALK− ALCL)

#### 2.2.1. Epidemiology and Clinical Features

ALK− ALCL is defined by the current WHO classification as a CD30+ PTCL lacking expression of the ALK protein, yet it is otherwise morphologically indistinguishable from ALK+ ALCL [9]. Systemic ALCL accounts for approximately 2–3% of NHLs (12% of PTCL), of which ALK− ALCL constitutes 15–50% of cases. This disease predominantly occurs in adulthood (median age 55–60 years), with a slight male predilection [12]. The majority of cases present with nodal disease, while a smaller subset shows extranodal involvement (20%), the most common sites including liver, lung, and skin (the latter as secondary cutaneous involvement) [9,13]. High stage (III–IV) disease at onset and systemic B-symptoms are frequent; BM is involved in about 7% of cases [13]. Similarly to other PTCLs, CHOP is the standard treatment. Despite early response to chemotherapy, relapse is fairly common [13] (Supplementary Table S1).

#### 2.2.2. Histological Findings and Immunophenotype

**Figure 3:**
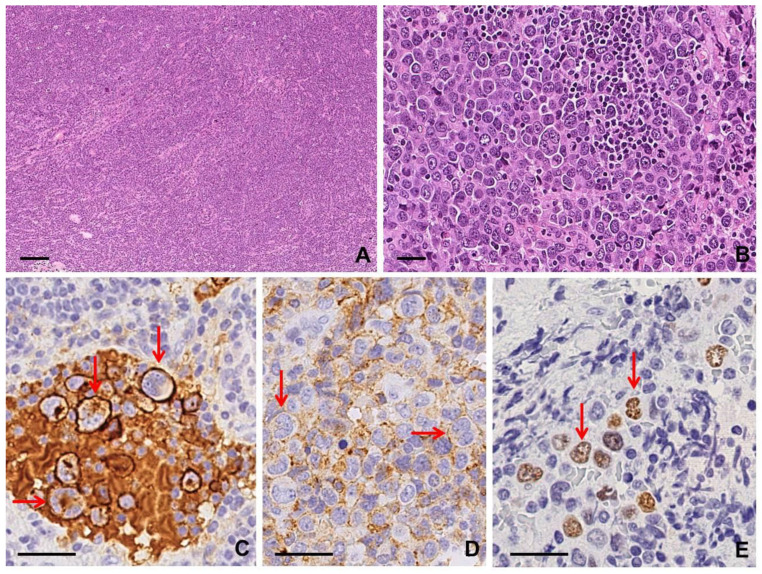
ALK− ALCL: (**A**) Diffuse pattern of growth in nodal ALK− ALCL (hematoxylin/eosin, 5×; scale bar: 100 µm). (**B**) RSLCs in a polymorphic infiltrate comprising small lymphocytes and eosinophils (hematoxylin/eosin, 30×; scale bar: 50 µm). RSLCs (red arrows) with expression of CD30 (**C**), positivity for CD4 (**D**) and for MUM1/IRF4 (**E**) (immunostaining, 40×; scale bar: 50 µm).

Nodal disease typically shows diffuse involvement by large neoplastic cells with pleomorphic nuclei, including the characteristic “hallmark” cells, similar to ALK+ ALCL; sinusoidal infiltration may be prominent, mimicking metastatic carcinoma [9,12]. The neoplastic cells are strongly and diffusely CD30 positive, with a membranous and Golgi pattern, but ALK negative. Most cases express T cell antigens; a cytotoxic phenotype (i.e., granzyme B+, perforin+) is frequently seen. Loss of T cell markers occurs more frequently than in other PTCLs, with a “null-cell” phenotype showing minimal expression of T cell lineage markers [13]. Expression of CD43, CD45/LCA, clusterin, and CD56 may be helpful in these cases. EMA expression is seen in a subset of cases [9,12]. Sclerosis and eosinophilia are occasionally present as well as RSCLs and their presence requires a careful investigation to exclude cHL [13] (Supplementary Table S1; Figure 3).

#### 2.2.3. Clues for Differential Diagnosis with cHL

In this context, the first approach is to recognize the different growth patterns of the neoplastic cells in the lymph node: sheet-like sinusoidal growth pattern of large pleomorphic cells in ALK− ALCL compared to isolated RSCs within an appropriate inflammatory background in cHL. Large cells with polylobate nuclei and/or “hallmark” cells may mimic RSCs and are also CD30+. However, RSC in cHL is CD15+, PAX5+, and CD45/LCA− [12,13] (Supplementary Table S1).

### 2.3. Breast Implant-Associated Anaplastic Large Cell Lymphoma (BIA-ALCL)

#### 2.3.1. Epidemiology and Clinical Features

BIA-ALCL was included as a new entity in the current WHO classification as a NHL characterized by a monoclonal population of large anaplastic cells which are uniformly CD30-positive and ALK negative and variably expressing T cell markers and EMA [9]. BIA-ALCL is considerably more frequent in women with textured implants and has been reported in association with both silicone gel and saline implants, either for cosmetic or reconstructive purposes [9,14]. It most frequently presents as a late-onset accumulation of seroma fluid between the implant and fibrous capsule in women without any reason for seroma formation, usually 8–10 years after implant [15]. Although it is generally considered an indolent disease, a subset of BIA-ALCL patients exhibit a more aggressive prognosis [14]. In this latter case, BIA-ALCL may present as a palpable tumor mass, with malignant cells infiltrating through the capsule and the surrounding tissue with a potential lymph node and systemic involvement; these patients have a 52.5% 2-year overall survival [16]. Regarding BIA-ALCL treatment, despite a lack of standardization, recent studies have emphasized the importance of complete surgical excision with removal of the implant, total capsulectomy, and removal of any mass with confirmation of negative margins, both for disease limited to the effusion and for infiltrative disease. Surgical excision of individual lymph nodes should be performed in cases suspicious for metastasis [17] (Supplementary Table S1).

#### 2.3.2. Histological and Cytological Findings and Immunophenotype

BIA-ALCL may be diagnosed on cytological examination of seroma fluid or histological evaluation of capsulectomy samples [9,18]. On cytological samples, medium to large atypical cells with abundant eosinophilic cytoplasm and irregular nuclei with prominent nucleoli are seen. These cells may sometimes have eccentric kidney-shaped nuclei or may be multinucleate with a RSLC morphology [18]. Usually, neoplastic cells count for up to 70% of the total fluid cellularity. On surgical samples, disease presenting as a seroma is composed of non-cohesive atypical neoplastic cells confined to the luminal side of the capsule, embedded in fibrinous material and with sparse inflammatory cells [16,18]. When presenting as a mass lesion, BIA-ALCL is characterized by sheets of malignant cells infiltrating the capsule and surrounding tissue, often with areas of necrosis and a variable acute inflammatory infiltrate, sometimes with prominent eosinophils [16]. BIA-ALCL neoplastic cells are strongly CD30 positive; EMA is positive in 43–90% of cases, while ALK is invariably negative [9,14]. Moreover, BIA-ALCL typically displays an incomplete T cell phenotype with variable loss of CD3, CD5, and CD7; most cases retain CD4 [18]. In very few cases, cells exhibit a NK/T cell phenotype with CD56 positivity and clonal rearrangement of the T cell receptor gene (TCR) [16]. Occasional cases have shown positivity for PAX5 and CD15, possibly leading to misdiagnosis as cHL; LMP1, however, is never expressed [16] (Supplementary Table S1).

#### 2.3.3. Clues for Differential Diagnosis with cHL

A correct diagnosis of BIA-ALCL may be achieved only through the evaluation of complete clinical, morphologic, immunophenotypic, and molecular data [9,18]. When presenting as a mass, BIA-ALCL RSLCs are positive for T antigens and EMA, while they do not express LMP1, PAX5, and CD15 [18] (Supplementary Table S1).

### 2.4. Angioimmunoblastic T cell Lymphoma (AITL)

#### 2.4.1. Epidemiology and Clinical Features

AITL is a mature T cell neoplasm, considered as the classic form of T-follicular helper derived (TFH) lymphoma, and accounts for 18.5% of all T and NK cell lymphomas [9]. AITL is an aggressive disease of elderly individuals characterized by generalized lymphadenopathy, hepatosplenomegaly, fever, effusion/ascites, and skin rash. In addition, autoimmune-like manifestations, including polyarthritis, have also been reported [19]. Laboratory tests exhibit immunological abnormalities, including hypergammaglobulinemia and positive Coombs test [19]. AITL has a poor prognosis, with a 5-year OS rate of approximately 30% [20]. Standard treatment strategies have not been established for AITL: Anthracycline-based CHOP or CHOP-like regimens are used as first treatment choice in the majority of cases [20] (Supplementary Table S1).

#### 2.4.2. Histological Findings and Immunophenotype

**Figure 4:**
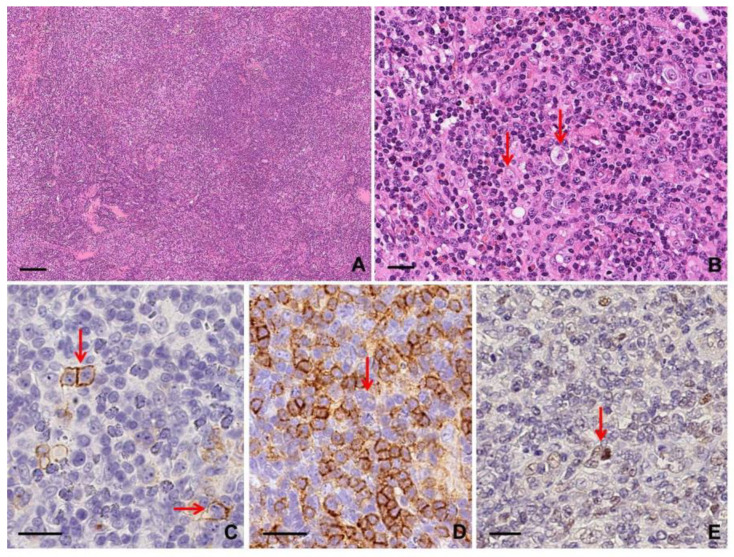
AITL: (**A**) Diffuse pattern of growth and marked proliferation of arborizing high endothelial venules in nodal AITL (hematoxylin/eosin, 5×; scale bar: 100 µm). (**B**) RSLCs (red arrows) in a polymorphic infiltrate comprising small lymphocytes, plasma cells, histiocytes, and eosinophils (hematoxylin/eosin, 30×, scale bar: 50 µm). RSLCs (red arrows) with expression of CD30 (**C**), negativity for CD4 in a context of small CD4+ lymphocytes (**D**) and positivity for BCL6 (**E**) in a context of small partially BCL6+ lymphocytes (immunostaining, 40×; scale bar: 50 µm).

Lymph nodes involved by AITL often show a complete architectural effacement with capsular and perinodal infiltration sparing the peripheral subcapsular sinus [9]. In most cases, cytological features of malignancy may not be evident because neoplastic T cells are typically outnumbered by reactive small lymphocytes, histiocytes, immunoblasts, eosinophils, and plasma cells [9,20]. A characteristic feature is a marked proliferation of arborizing high endothelial venules [19]. Neoplastic cells appear as small to medium-sized T lymphocytes, with round and slightly irregular nuclei and abundant pale cytoplasm, tending to cluster close to vessels: their identification is a critical clue for the diagnosis [19]. These cells are positive for TFH markers, such as CD4, CD3, CD2, and CD5; CD279 (PD-1, PDCD1), CXCL13, and BCL6 positivity is also observed. Conversely, expression of CD7 is less common and CD8 is negative [9,20]. A CD21+ CD23+ expanded follicular dendritic cell (FDC) network surrounding small vessels is usually present [9,20]. Large CD20+, PAX5+, and CD79α+ immunoblasts are commonly observed and may also co-express CD30 and CD15 [21]. They are usually, but not always, infected by EBV, showing positivity for EBER and LMP1 [20]. In some cases, these large B immunoblasts within the inflammatory background can mimic RSLCs and may lead to a misdiagnosis of cHL [5] (Supplementary Table S1; Figure 4).

#### 2.4.3. Clues for Differential Diagnosis with cHL

RSLCs in AITL represent EBV−positive immunoblastic proliferations driven by TFH neoplastic cells and, found in a cellular milieu reminiscent of mixed cellularity or lymphocyte-rich cHL, can be easily interpreted as RSCs. Histological clues of AITL are arborizing high endothelial venules together with an expanded CD21-positive follicular dendritic cell network and atypical medium-sized lymphoid cells with a TFH phenotype [5]. The presence of a CD4+, CD10+, BCL6+ T cell population strongly supports a diagnosis of AITL [19]. In AITL, RSLCs usually show an intense CD20 positivity, unlike RSCs in cHL showing a variable and often weak CD20 expression. Moreover, CD3 staining fails to recognize “pseudorosettes” around RSLCs in AITL. In cases of doubt, monoclonal or oligoclonal rearrangement of the TCR genes can be demonstrated in the vast majority of AITL cases [20] (Supplementary Table S1).

### 2.5. Follicular Peripheral T cell Lymphoma (F-PTCL)

#### 2.5.1. Epidemiology and Clinical Features

F-PTCL is a rare form of T cell lymphoma, without sex or age prevalence, primarily involving the follicles; it may morphologically resemble follicular lymphoma of B cell origin [9]. The clinical features overlap with AITL [20]. A subset of patients shows long-term survival despite multiple relapses; the prognosis might be slightly better than that of AITL [19] (Supplementary Table S1).

#### 2.5.2. Histological Findings and immunophenotype

**Figure 5:**
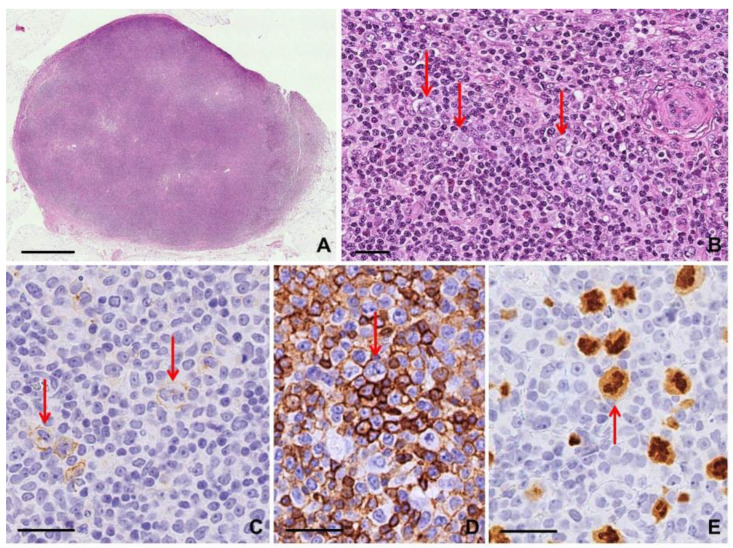
F-PTCL. (**A**) Nodular pattern of growth in nodal F-PTCL (hematoxylin/eosin, 0.5×; scale bar: 0.5 cm). (**B**) Isolate RSLCs (red arrows) in a context of small lymphocytes (hematoxylin/eosin, 30×; scale bar: 50 µm). RSLCs (red arrows) weakly positive for CD30 (**C**), strongly positive for CD45/LCA (**D**), and for PAX5 (**E**) (immunostaining, 40×; scale bar: 50 µm).

Two different growth patterns can be recognized in lymph node involvement by F-PTCL: a true follicular pattern, mimicking follicular lymphoma (FL-like), or, more commonly, a progressive transformation of germinal center-like pattern (PTGC-like) [20]. In FL-like F-PTCL, the neoplastic cells form intrafollicular aggregates sustained by a meshwork of FDC. The neoplastic cells are, by definition, positive for at least two T follicular helper cell markers: CD3+, CD4+, PD1+, ICOS+, BCL6+, and CD10+ [9,22]. A component of large EBV+/− and CD45/LCA+ immunoblastic B cells is often identified, often with RSLC morphology and immunophenotype [20,22] (Supplementary Table S1; Figure 5).

#### 2.5.3. Clues for Differential Diagnosis with cHL

F-PTCL can strongly mimic lymphocyte-rich cHL for the presence of a B cell-rich microenvironment, with RSLCs CD20+ in 32% of cases and LMP1+ in 47% of cases, with CD30, CD15 and PAX5 co-expression in most cases [22]. Both F-PTCL and lymphocyte-rich cHL frequently present B symptoms and generalized lymphadenopathy at onset, and morphologically show a nodular growth pattern. Lymphocyte-rich cHL is more frequently found in the Waldeyer’s ring and in cervical lymph nodes, in contrast to the frequent inguinal localization of F-PTCL, the latter being associated with older age, higher stage at diagnosis, and a more dismal prognosis [5,11]. Another feature common to cHL and F-PTCL is the presence of rosetting T cells around RSCs/RSLCs [22,23]. Monoclonal neoplastic proliferation of T cells with TFH immunophenotype is indicative of F-PTCL [20,22,23] (Supplementary Table S1).

## 3. B cell Lymphomas, High Grade

### 3.1. Diffuse Large B Cell Lymphoma, Not Otherwise Specified (DLBCL, NOS)

#### 3.1.1. Epidemiology and Clinical Features

DLBCL is a neoplasm comprised of large B cells, arranged in a diffuse growth pattern [9], and is the most common type of NHL with an incidence rate of 6.3% [24,25]. DLBCL is more prevalent in elderly patients (7th decade), although it may occur in young adults and rarely in children, with a slight male predominance [9,25]. Clinically, most patients present with a rapidly growing tumor mass involving one or more lymph nodes and extranodal sites; approximately 40% of patients present with extranodal disease, most commonly localized in the gastrointestinal tract [24,25]. About one-third of patients with DLBCL present with B symptoms at onset along with symptoms related to organ involvement [25]. Serum lactase dehydrogenase (LDH) and beta-2-microglobulin are often increased, and approximately half of patients present at early stage (I–II), whereas the other half presents with an advanced stage (III–IV) disease [9,24]. BM is involved in about 10–20% of cases, either in a concordant (BM involved by DLBCL) or discordant form (BM involved by low-grade B cell lymphoma) [24].

DLBCL is a highly heterogeneous group of neoplasms with a variable clinical course and prognosis [9,24]. R-CHOP protocol is the standard therapy; however, about 30–40% of patients will relapse or will be refractory to treatment [24]. Autologous stem cell transplantation is often recommended for younger patients [24]. Prognosis of patients with relapse or who cannot undergo transplantation (elderly patients or with comorbility) is very poor [24] (Supplementary Table S2).

#### 3.1.2. Histological Findings and Immunophenotype

**Figure 6:**
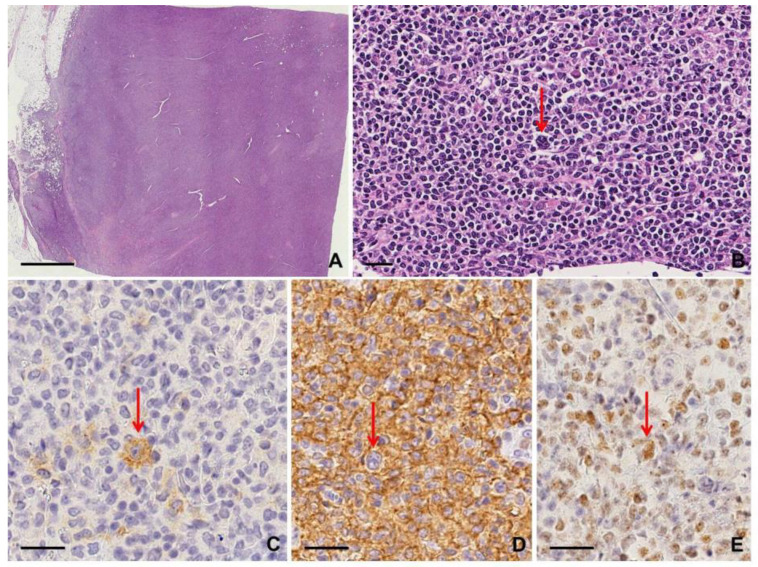
DLBCL, NOS: (**A**) Diffuse pattern of growth in nodal DLBCL, NOS (hematoxylin/eosin, 5×; scale bar: 0.5 cm). (**B**) RSLCs (red arrows) in a context of medium-to-large lymphocytes (hematoxylin/eosin, 30×, scale bar: 50 µm). RSLCs (red arrows) with expression of CD30 (**C**), positivity for CD20 in a context of CD20+ medium-to large lymphocytes (**D**) and positivity for BCL6 in a context of BCL6+ medium-to large lymphocytes (**E)** (immunostaining, 40×; scale bar: 50 µm).

DLBCL shows large- to medium-sized neoplastic cells, with a diffuse growth pattern totally or partially effacing normal nodal or extranodal architecture [9,24]. Fine fibrosis, sclerosis, geographic necrosis, or a combination can be observed. Single-cell apoptosis can be prominent and the mitotic rate may be high [9,24]. Variable numbers of background reactive small T cells and histiocytes are present in all cases of DLBCL. Several DLBCL variants have been described, the centroblastic, immunoblastic, and anaplastic variants being the most represented [9,24]. The anaplastic variant is the less common (3% of all DLBCLs cases) and is characterized by large or very large neoplastic cells with pleomorphic or bizarre nuclei, mimicking RSLCs or ALCL cells [9,24]. This variant often has a partial or extensive sinusoidal growth pattern. DLBCLs express pan B cell antigens such as CD19, CD20, and CD22 as well as B cell transcription factors including PAX5, BOB1, and OCT2 [9,24]. About 50–70% of cases express surface or cytoplasmic immunoglobulin (IgM or, less frequently, IgG and IgA). However, at least one-third of DLBCL cases are negative for Ig and rarely lack one or more pan B cell antigens with negativity for pan T cell antigens [9,24]. About 10–15% of cases of DLBCL are positive for CD30 and about 20–25% of cases are positive for PD-L1 [9,24]. Conventional cytogenetic analysis is helpful in the work-up of DLBCL for a global view of chromosomal abnormalities [24] (Supplementary Table S2; Figure 6).

#### 3.1.3. Clues into Differential Diagnosis with cHL

First of all, the lack of an appropriate background or an extranodal localization argue against a cHL. Differential diagnosis can be problematic when DLBCL presents as an anaplastic variant, especially compared to lymphocyte-depleted cHL [5,24]. In order to differentiate RSC from RSLC, immunohistochemistry is of pivotal importance. B cell markers are very rarely expressed in cHL [9]. MUM1/IRF4 is a very sensitive marker for RSC in cHL, while CD45/LCA positivity is indicative of RSLC in DLBCL [5]. Moreover, BOB1 positivity is reported in >50% of RSCs, whereas OCT2 expression may occur less frequently; co-expression of both BOB1 and OCT2 in cHL is extremely rare, unlike in DLBCL RSCLs [5] (Supplementary Table S2).

### 3.2. T Cell/Histiocyte-Rich Large B Cell Lymphoma (THRLBCL)

#### 3.2.1. Epidemiology and Clinical Features

THRLBCL is a large B cell lymphoma (LBCL) characterized by sparse neoplastic cells, usually single and not forming aggregates or sheets [9]. The background is made of reactive T lymphocytes and histiocytes with very few small, non-neoplastic, B lymphocytes. By definition, THRLBCL is EBV/LMP1 negative [9].

It is a rare subtype representing < 5% of all DLBCLs cases with a median age of onset of 49–57 years [26]. Clinical manifestations include B symptoms and hepatosplenomegaly, with common BM involvement; more than half of patients show advanced stage disease at onset [9,27]. Patients’ response to R-CHOP therapy is poor with a 3-year OS ranging from 46% to 72% [27] (Supplementary Table S2).

#### 3.2.2. Histological Findings and Immunophenotype

THRLBCL is characterized by a diffuse growth pattern; neoplastic cells constitute < 10% of total cellularity and show centroblastic, immunoblastic, and Hodgkin-like/RSLC morphology [9,27]. Host T lymphocytes, with or without non-epithelioid histiocytes, are major components of the neoplasm. The lymphoma cells express pan B cell markers (CD20, CD79α, PAX5), PD-L1 (most cases), BCL6 (50–90%), c-MYC (65%), BCL2 (40%), EMA (30%), and CD10 (in a minority) [26]. CD5, CD15, CD30, and CD138 are usually negative. Follicular dendritic cell (FDC) meshworks are absent in THRLBCL [28]. EBV/LMP1 is, by definition, negative [9] (Supplementary Table S2).

#### 3.2.3. Clues into Differential Diagnosis with cHL

Overlapping morphological features and molecular profiling between THRLBCL and NLPHL and cHL are well known [28]. Moreover, it has been showed that some patients with THRLBCL have a prior history of NLPHL [26]. For these reasons, differential diagnosis can be extremely difficult [28]. Currently, evidence of any areas of nodularity in the neoplasm supports NLPHL, while a diffuse pattern is in favor of THRLBCL [26]. PD-1-positive T cells may be observed in THRLBCL, but, unlike in lymphocyte-rich cHL, there is not a rosette configuration surrounding tumor cells and the background is more monotonous than in cHL, lacking eosinophils. Unlike in cHL, typically showing downregulation of B cell markers, in THRLBCL RSLCs are strongly CD20-positive and lack CD30, CD15, and EBV expression [26] (Supplementary Table S2).

### 3.3. ALK-Positive Large B cell Lymphoma (ALK+ LBCL)

#### 3.3.1. Epidemiology and Clinical Features

ALK+ LBCL is an ALK-positive lymphoma with a plasma cell-like immunophenotype, accounting for less than 1% of all cases of DLBCLs [9]. It is more common in young men but can arise in all age groups (range: 9–85 years), including children in one-third of cases. No ethnic predisposition is documented [29]. Clinically, diffuse lymphadenopathy is common and half of patients have B symptoms [29]. Extranodal sites of involvement include mediastinum, bones, nasopharynx, tongue, stomach, liver, spleen, and skin [9]. Approximately 60% of patients present with advanced stage disease and 25–30% have BM involvement [9,29]. ALK+ LBCL shows a very aggressive clinical behavior, with a high relapse rate and poor response to standard treatment with CHOP or CHOP derived regimens [29]. The 5-year OS is 34%, but patients younger than 35 years and with low-stage disease have a better prognosis than those with advanced disease (about 1 year) [26,29] (Supplementary Table S2).

#### 3.3.2. Histological Findings and Immunophenotype

ALK+ LBCL involves lymph nodes in a diffuse or sinusoidal pattern; a mixed pattern can also occur [9]. The neoplastic B cells are monomorphic, with immunoblast or plasmablast-like features showing round nuclei, prominent central nucleoli, and abundant basophilic cytoplasm; multi-nucleation and anaplastic features, both mimicking RSLCs, with necrosis can be observed [9,26]. By definition, the neoplastic cells are ALK positive and, in a small subset of cases, they may more closely resemble mature plasma cells [9,26]. Plasma cell-associated markers are usually positive, including CD138, VS38, and MUM1/IRF4 [9]. BOB1 and/or OCT2 are usually positive, while CD10 and BCL6 are usually negative [29]. Other pan B cell and pan T-markers are mostly negative, although 40–50% of neoplasms can show partial CD4 positivity, and rarely CD57, CD43, or perforin may be also positive [9,26]. Approximately 90% of cases express EMA, whereas 10% can be positive for pan cytokeratin with a dot-like paranuclear pattern. More than 95% of cases are positive for IgA with light chain restriction [9,26]. CD30 is negative, with rare exceptions [9] (Supplementary Table S2).

#### 3.3.3. Clues for Differential Diagnosis with cHL

The first useful approach, as always, is a morphological evaluation: in ALK+ LBCL, the vast majority of cells are neoplastic, unlike cHL; staining for Ig, CD30, and ALK can be very helpful in order to properly recognize RSLCs [26]. Moreover, CD3 stain fails to demonstrate rosettes around RSLCs [9] (Supplementary Table S2).

### 3.4. Primary Mediastinal (Thymic) Large B Cell Lymphoma (PMBL)

#### 3.4.1. Epidemiology and Clinical Features

PMBL is a neoplasm arising from thymic B cells with distinctive clinical, pathological, and molecular features, which account for 2–3% of all NHLs and 6–10% of all DLBCLs [9]. The median age is 35–37 years with female/man ratio of up to 2:1 [30]. Patients present with an enlarging and bulky (>10 cm diameter) anterior/superior mediastinal mass [9,26]. Compressive and infiltrative symptoms, including superior vena cava syndrome and dyspnea, are common and approximately 80% of patients present with early stage disease [30]. Cervical and supraclavicular lymphadenopathy is often documented but systemic symptoms are present in less than 20% of cases with rare BM involvement [26]. Elevated serum LDH and low serum beta-2 microglobulin levels are frequent [26]. The 5-year OS is 70–85% [30]. Due to the rarity of this disease, the optimal therapy has not been defined. However, recent studies have shown that regimens integrating rituximab into intensive chemotherapy might yield a better outcome [30] (Supplementary Table S2).

#### 3.4.2. Histological Findings and Immunophenotype

The characteristic feature of PMBL is a diffuse growth pattern of medium to large-sized neoplastic cells surrounded by a variable degree of sclerosis and collagen band (compartmentalization) mimicking nodules [9,31]. The lymphoma cells have, characteristically, round or pleomorphic nuclei and clear, pale, or slightly basophilic cytoplasm [9,31]. RSLCs may be observed [26].

**Figure 7:**
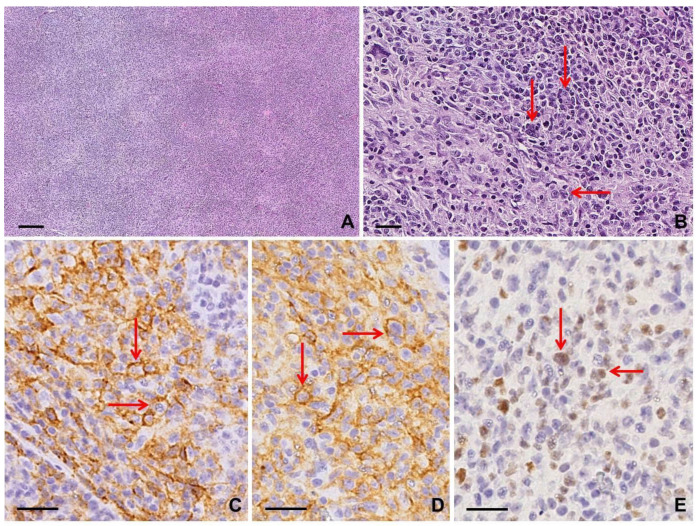
PMBL: (**A**) Diffuse pattern of growth in PMBL (mediastinal biopsy; hematoxylin/eosin, 5×; scale bar: 0.5 cm). (**B**) RSLCs (red arrows) in a polymorphic infiltrate comprising lymphocytes, histiocytes, in a context of sclerosis (hematoxylin/eosin, 30×; scale bar: 50 µm). RSLCs (red arrows) with expression of CD30 (**C**), positivity for CD23 in a context of CD23+ medium-to large lymphocytes (**D**), and for BCL6 (**E**) (immunostaining, 40×; scale bar: 50 µm).

Pan B cell antigens, as well as CD10 (25%), BCL6 (95%), MUM1/IRF4 (95%), c-MYC (65%), fascin (30%), and BCL2 (65%), are expressed in neoplastic cells [31]. CD23 is characteristically positive in 70–95% and CD30 in approximately 80% of cases [9,31] (Figure 7).

#### 3.4.3. Clues for Differential Diagnosis with cHL

Distinguishing between cHL involving the mediastinum and PMBL can be challenging, but it is crucial for the different first-line treatment and outcome [5,31]. Both entities commonly affect young adults with a slight female predominance; they are of B cells origin and share the presence of large neoplastic cells, RSCs and RSLCs, respectively, in a fibrotic or sclerotic stroma, whose immunophenotype is overlapping (Ig−, CD30+) [9,31]. However, unlike cHL, CD30 is less intensely and variably expressed in the PMBL large cells [5]. Typical PMBL neoplastic cells express CD20, CD23, CD45/LCA, CD79α, and OCT2/BOB1 [5,9,31]. Conversely, in cHL CD20 and CD79α are negative or weakly expressed on a subset of the neoplastic cells while CD15 is rarely observed in PMBL. Both CD15 and EBV expression provide high sensitivity for cHL in this differential diagnostic context. CD23 and p63 are also very useful, with a high positive predictive value (98% and 96%, respectively) for PMBL [5,31] (Supplementary Table S2).

### 3.5. Mediastinal Gray-Zone Lymphoma (GZL)

#### 3.5.1. Epidemiology and Clinical Features

GZL is an uncommon lymphoma with an intermediate clinical, morphological, and immunophenotypical profile between DLBCL and cHL [9]. GZL most commonly affects young men (median age at diagnosis within the third decade) as a large anterior mediastinal bulky lesion (≥10 cm) with or without extra-mediastinal involvement [9,32]. Patients presenting without mediastinal disease tend to be older (median age within the fifth decade) [32,33]. Extra-mediastinal involvement includes supraclavicular and cervical lymphadenopathy with peripheral and intra-abdominal lymphadenopathy. Other organs may be involved, such as lung by direct extension, as well as liver, spleen, and rarely BM [9,33].

GZL is an aggressive tumor, whose first-line treatment is CHOP. Thus, distinguishing GZL from cHL and PMBL is clinically relevant [9,31] (Supplementary Table S2).

#### 3.5.2. Histological Findings and Immunophenotype

Wide variation in the morphological spectrum is characteristic of GZL: about two-thirds of cases show RSLCs, whereas others demonstrate sheets of large cells closely resembling DLBCL or PMBL with compartmentalizing sclerosis [32]. An important morphological feature of GZL is its high tumor cell content, with neoplastic cells occurring in confluent sheets in a background containing scarce inflammatory cells, although eosinophils, histiocytes, and small lymphocytes can be seen. Tumor cells nuclei show a great variability in size and shape with infrequent eosinophilic nucleoli compared to RSCs in cHL. In GZL tumor cells show centroblastic or immunoblastic appearance and marked pleomorphism [33,34]. Variable fibrosis can be present, including extensive coarse fibrosis as well as fine compartmentalizing fibrosis [9,34].

All GZLs show expression of at least one B cell marker but, similarly to morphologic findings, the immunophenotype of GZL is variable with transitional and divergent patterns [34]. Tumors with RSLCs morphologically resembling cHL may express prominent CD20, weaker/absent CD30, and absent CD15, whereas tumors resembling PMBL are frequently strongly positive for CD30 and CD15 while negative for CD20 and CD79α [9,34]. MUM1/IRF4 is moderately positive in most cases, whereas it remains controversial whether EBV-positive cases should be defined GZL or should be included in other EBV-related entities [34] (Supplementary Table S2).

#### 3.5.3. Clues for Differential Diagnosis with cHL

GZL lacks nodular growth pattern and fibrous bands, although a variable degree of fibrosis may be seen [9]. Nodular sclerosis cHL is expected to show typical nodular pattern with prominent sclerosis. In contrast to cHL, in GZL areas of necrosis are often scarce and lacking neutrophilic infiltrate [32,33]. Furthermore, neoplastic cells in GZL exhibit nuclei with a broader range in size and shape and more infrequent eosinophilic nucleoli, than RSC and its variants in cHL [33,34].

In cHL, RSCs typically show variable downregulation of B cell markers such as CD20, PAX5, OCT2, and BOB1, and strong CD30 and CD15 expression [32]. In GZL, RSLCs are usually CD20 strongly positive, lack CD15 with weak or absent CD30 (Supplementary Table S2).

### 3.6. Lymphomatoid Granulomatosis (LG)

#### 3.6.1. Epidemiology and Clinical Features

LG is an angiocentric and angiodestructive lymphoid infiltrate composed of EBV positive B cells typically involving extranodal sites, usually the lungs [9]. The median age at onset is 46–48 years with a male predominance, and it is associated with impaired immune function, including Wiskott–Aldrich syndrome, HIV infection, high-dose chemotherapy, and transplantation [9,26]. The lung is nearly always involved, and patients present with multiple bilateral pulmonary nodules, mainly in the lower lobes, or rarely with an interstitial and/or reticulonodular pattern [9,35]. Fever and cough are the most common symptoms. Patients may have coexisting involvement of the brain and peripheral nervous system, skin, kidneys, liver, gastrointestinal tract, and upper respiratory system [26,35]. The BM, lymph nodes, and spleen are rarely affected. Outcome of patients treated with steroids and/or chemotherapy is poor (median OS of 14 months); in a prospective clinical trial, patients with 1–2 LG grades have been treated with interferon-α, while those with grade 3 LG were treated with immunochemotherapy [35] (Supplementary Table S2).

#### 3.6.2. Histological Findings and Immunophenotype

Angiocentric and angiodestructive infiltrate of lymphocytes (including atypical cells) and histiocytes is the hallmark of LG [9]. Large atypical cells with immunoblastic to Hodgkin-like/RSLC features are variably seen ranging from sparse cells to confluent sheets [9,35]. Occasional plasma cells may be seen in the background, while neutrophils and eosinophils are absent. Necrosis is variably seen and it is more pronounced in high-grade lesions. Granulomas are not present in most cases. The large atypical cells are immunoreactive for pan B cell markers such as CD20, CD79α, and PAX-5. CD30 is often positive and CD15 is negative [9,26]. T cells are CD3- and CD4-positive. EBV latency is type III (LMP1+ and EBNA2+) in most cases [9,31] (Supplementary Table S2).

#### 3.6.3. Clues for Differential Diagnosis with cHL

Angiocentric and angiodestructive patterns and extranodal localization are not a feature of cHL [9]. Histologically, LG is made of large atypical immunoblastic-like B cells, which are uncommon in cHL [26,35] (Supplementary Table S2).

### 3.7. Primary Effusion Lymphoma (PEL)

#### 3.7.1. Epidemiology and Clinical Features

PEL is a Human Herpes Virus 8 (HHV8)-driven neoplasm involving pleural, pericardial, or peritoneal cavities without mass-forming [9]. Extracavitary PEL refers to a solid tumor with similar features as conventional PEL [9]. The median age at diagnosis is 45 years [36]. Most patients are immunosuppressed (HIV or post-transplant related); up to two-thirds of PEL patients show concurrent or previous Kaposi sarcoma and about one-third have had Castleman Disease (CD) [9,33]. Rarely, PEL patients do not have a history or evidence of immunosuppression, and only one case of PEL metachronous to CD in an immunocompetent patient has been reported [37]. Patients with conventional PEL present with effusion-related symptoms along with B symptoms in most cases [25,26]. Prognosis of PEL remains poor with a median OS of less than 1 year [36]. No standard therapy exists due to its low incidence. CHOP-like regimens alone or associated with high-dose methotrexate have been proposed [36] (Supplementary Table S2).

#### 3.7.2. Histological Findings and Immunophenotype

**Figure 8:**
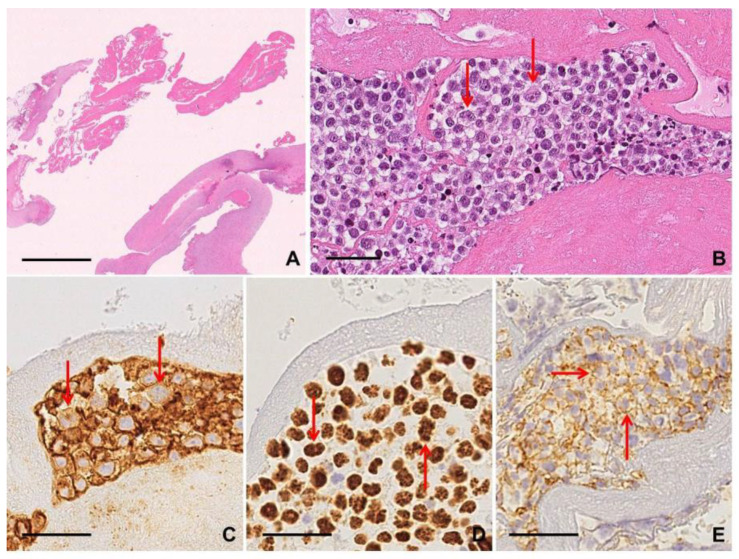
PEL: (**A**) Small cellular aggregates in fibrinoid background with hypocellularity (peritoneal biopsy; hematoxylin/eosin, 5×; scale bar: 0.5 cm). (**B**) RSLCs (red arrows) in small aggregates of neoplastic cells (hematoxylin/eosin, 30×; scale bar: 50 µm). RSLCs (red arrows) with CD30 expression (**C**), strong HHV8 positivity (**D**), and CD45/LCA expression (**E**) in a context of medium-to large lymphocytes (immunostaining, 40×; scale bar: 50 µm).

Cytology smears or cell block sections of effusion specimens show isolated pleomorphic tumor cells resembling immunoblasts, plasmablasts, or anaplastic cells with Hodgkin-like/RSLC featuring prominent nucleoli and moderate to abundant basophilic cytoplasm [9,26,37]. The lymphoma cells are positive for HHV8 and often for MUM1/IRF4 (up to 100%), CD38 (up to 100%), CD45/LCA (>80%), CD138 (35–75%), and CD30 (>50%). EMA and cytoplasmic immunoglobulin can be positive as well. BOB1 and/or OCT2 are usually positive and may be helpful to establish B cell lineage. Aberrant T cell antigen expression can be observed, including CD45RO, CD7, and CD4; CD10, CD15, and BCL6 are negative [36]. In situ hybridization for EBER is often positive, especially in the HIV setting. Extracavitary PEL more often than not expresses CD20 or CD79α and is less likely to be positive for CD45/LCA or CD138 [26,36] (Supplementary Table S2; Figure 8).

#### 3.7.3. Clues for Differential Diagnosis with cHL

Only extracavitary PEL may show features resembling cHL. In the adequate morphological context, HHV8 immunostaining is specific for PEL; RSCs in cHL are usually CD45/LCA-, CD138-, OCT2- unlike RSLCs in PEL [9,26,36] (Supplementary Table S2).

### 3.8. EBV-Positive Diffuse Large B Cell Lymphoma, Not Otherwise Specified (EBV+ DLBCL, NOS)

#### 3.8.1. Epidemiology and Clinical Features

EBV+ DLBCL, NOS shows a diffuse grow pattern of large malignant EBER positive cells [9,38]. Patients with EBV-positive DLBCL, NOS lack a history of immunodeficiency or previous lymphoma [9,39]. Clinically, patients are usually, but not exclusively, diagnosed at older age and extranodal involvement is frequent (mainly gastrointestinal tract, skin, BM) [9]. A higher proportion of patients show elevated LDH levels, advanced clinical stage, and worse performance status [38,39]. EBV+ DLBCL, NOS is an aggressive lymphoma with OS ranging from 17 to 36 months [38]. There is not a standard approach and treatment options are usually in accordance with current strategies for DLBCL, NOS [38] (Supplementary Table S2).

#### 3.8.2. Histological Findings and Immunophenotype

EBV+ DLBCL, NOS has a variable histological aspect depending on the tumor cell density and the inflammatory background [9,39]. The polymorphous subtype is the most frequent and it is constituted by medium to large-sized, scattered neoplastic RSLCs in a reactive background made of small lymphocytes, histiocytes, and plasma cells [39]. Other cases show a monomorphic proliferation of large neoplastic cells with immunoblastic or centroblastic morphology resembling DLBCL, without the inflammatory background [9,39]. Angiocentric/angiodestructive lesions can be present, with extensive coagulative geographical necrosis [39].

The neoplastic cells characteristically express pan B cell markers such as CD19, CD20, CD22, and CD79α, and more frequently show an activated B cell phenotype expressing MUM1/IRF4, with CD10 and BCL6 negativity [9,38]. CD30 is positive in 40% of cases, with a poorer prognosis in elderly patients, while few cases are CD15-positive [38].

In situ hybridization for EBV is mandatory for the diagnosis [9]. LMP1 is positive in most cases (76%), while EBNA-2 is expressed only in a minority of cases (14%) [38,39] (Supplementary Table S2).

#### 3.8.3. Clues for Differential Diagnosis with cHL

The presence of RSLCs within a reactive background made of small lymphocytes and histiocytes in the polymorphous subtype of EBV+ DLBCL, NOS needs to be differentiated from cHL [39]. Angiocentric, angiodestructive lesions, and extensive coagulative necrosis is typical of EBV+ DLBCL; moreover, strong immunohistochemical expression of B cell lineage markers is characteristic of EBV+ DLBCL while it is very unusual in cHL [9]. Clinical context (extranodal involvement and older age at onset) can be helpful too to reach the correct diagnosis [38,39] (Supplementary Table S2).

### 3.9. Mantle Cell Lymphoma (MCL)

#### 3.9.1. Epidemiology and Clinical Features

MCL comprises approximately 5% of NHLs and usually manifests as generalized lymphoadenopathy with male predilection and median age at onset of 66 years [9,40]. Extranodal disease is very common, usually involving peripheral blood, spleen, gastrointestinal tract, and, in less than 5% of cases, the central nervous system [41]. According to the current WHO classification, MCL diagnosis is based on its distinctive morphology and the presence of the characteristic chromosomal translocation t(11;14) (q13;q32) that juxtaposes the *CCND1* gene to the immunoglobulin heavy chain (IGH) gene, thus resulting in constitutive overexpression of CyclinD1 [9]. Despite its aggressive clinical course, not all patients require immediate treatment: asymptomatic patients may benefit from expectant management. For patients who require treatment, a combination of Rituximab, hyperfractionated cyclophosphamide, vincristine, doxorubicin, dexamethason (R-Hyper CVAD) alternating with Rituximab, high dose methotrexate and cytarabine (R-MA) is used [41] (Supplementary Table S2).

#### 3.9.2. Histological Findings and Immunophenotype

Classical MCL may show a vaguely nodular, diffuse, or mantle zone growth pattern where the tumor cells surround preserved germinal centers as expanded mantle zones [9,40]. The tumor cells are uniform, usually small- to medium-sized with irregular nuclear contours, resembling centrocytes [9]. The less common blastoid and pleomorphic variants, associated with a more aggressive prognosis, may be present at diagnosis or during disease progression [9,40]. Neoplastic B cells are typically positive for CD19, CD20, CD22, CD79α, CD43, CD5, and FMC7, whereas CD23, CD10, CD200, and BCL6 are negative [9,42]. CyclinD1 immunostain is positive in 95% of cases; SOX11 expression, positive in 90–95% of MCL and negative in non-malignant lymphocytes and CLL/SLL, may be useful when CyclinD1 is negative [9,40,41]. Demonstration of t(11;14), mainly in CyclinD1-negative cases, is generally required to confirm MCL diagnosis [9,42] (Supplementary Table S2).

Presence of RSLCs in MCL is a very rare, but well-known, occurrence [43]. RSLCs in MCL invariably express CD30 and variably express CD15. In some cases, weak and partial expression of CD20+ and CD45/LCA+ is reported as well as EBV positivity. A spectrum of background ranging from minimally associated inflammatory cells to a cHL-like mixed inflammatory population is reported [43]. Moreover, combined MCL and cHL lymphoma is also described and a divergent clonal evolution from a common precursor has been hypothesized [43,44], leading to RSLC in MCL and/or RSC in cHL combined with MCL because of EBV infection, acquisition of additional genetic aberrations such as *PD-L1/2* amplification, and certain mutations such as *TP53* [44,45] (Supplementary Table S2).

#### 3.9.3. Clues into Differential Diagnosis with cHL

On the basis of the histological growth pattern in neoplastic tissue and clonal relationship of RSLC in MCL and RSC in composite lymphoma, we can recognize two entities [43]: in the first variant, RSLCs are present in *recurrent* MCL specimens as single cell and small clusters among MCL cells that retain a vague nodular pattern [43,45]. RSLCs are frequently associated with small clusters of histiocytes and might be surrounded by T cell rosettes; however, eosinophils, neutrophils, and plasma cells are usually absent [43]. Clonal relationship with identical t(11;14) rearrangements in RSLC and MCL cells has been proven, thus advancing the hypothesis that RSLC is not an early event in MCL but rather a transformed event after a longstanding disease [46]. These cases must be distinguished from true lymphocyte-rich cHL in which RSCs are located among the expanded, morphologically, and immunohistochemically benign mantle zone cells [43].

The second pathological variant includes cases of *true* composite lymphoma with distinct, separate MCL and cHL components [43]. In this variant, nodules of cHL are intermixed with diffuse or nodular areas of typical MCL. The cHL and MCL populations have been demonstrated to be different clones by clonal analysis of the microdissected cells, suggesting that separate components arise from different clones [47].

The prognosis of these two entities is unknown due to their rarity; the first lesion may be approached by using a NHL/MCL regimen, whereas in composite lymphoma a tailored approach containing chemotherapy sensitive for both MCL and cHL seems to be the best option [43] (Supplementary Table S2).

## 4. B Cell Lymphomas, Low Grade

### 4.1. Follicular Lymphoma (FL) 

#### 4.1.1. Epidemiology and Clinical Features

FL is the second most common subtype of NHLs, accounting for 20–25% of all NHLs [48]. The genetic hallmark of FL is the t(14;18)(q32;q21) translocation and its variations that juxtapose the BCL2 and IGH genes, leading to the overexpression of the antiapoptotic protein, BCL2 [9]. FL is slightly more common in men than in women, and it is more common in older people, with a median age range of 60 to 65 years at the time of diagnosis [9,48]. FL presents with enlarged lymph nodes in the neck or abdomen with B symptoms [9]. Despite the fact that patients with FL are frequently asymptomatic, the majority of them present with advanced-stage disease, which is considered incurable but associated with long median survival. An alternative therapy to the wait-and-see strategy is to treat these patients with single-agent rituximab [49]. Only 20–30% of patients present with stage I–II disease: early-stage FL is treated with external beam radiotherapy with or without systemic therapy, which imparts excellent disease control leading to long-term complete remission in ~50% of patients [48] (Supplementary Table S3).

#### 4.1.2. Histological Findings and Immunophenotype

FL is characterized by a follicular/nodular growth pattern and a variably represented diffuse neoplastic component effacing the normal lymph node architecture [9]. Neoplastic follicles typically are round and homogeneous in appearance, infiltrating the lymph node capsule and extending into the perinodal adipose tissue [9,48]. Neoplastic follicles are made up of small to medium-sized B cells, derived from germinal-center B cells, with a cleaved shape (centrocytes) and larger, non-cleaved B cells (centroblasts) which have a moderate amount of cytoplasm [9]. These tumor cells are admixed with reactive T cells, FDCs, and histiocytes, with occasional macrophages, granulocytes, and plasma cells [9,48]. The presence of centroblasts is used to determine the grade of FL according to the WHO criteria [9]. Neoplastic B cells in FL are CD20+, CD23+/−, CD43−, CD10+, BCL2+, BCL6+, CD3−, CD5−, and CyclinD1− [9]. In difficult cases, molecular and cytogenetic tests are also required, which include immunoglobulin gene rearrangements (clonality testing) and *BCL2* translocation (t(14;18) or variants) by FISH [48].

**Figure 9:**
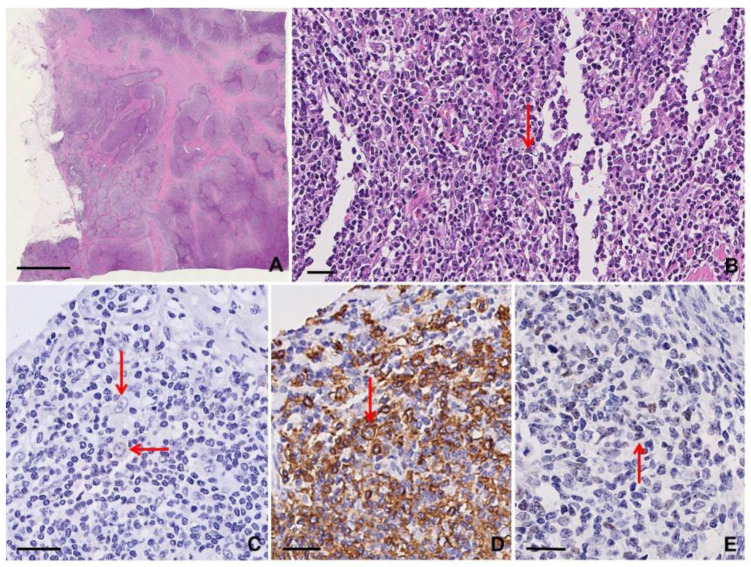
FL: (**A**) Nodular pattern of growth with fibrosis in nodal FL (3b) (hematoxylin/eosin, 0.5×; scale bar: 0.5 cm). (**B**) RSLCs (red arrows) in a typical context of a polymorphic infiltrate comprising small T lymphocytes, FDCs and histiocytes (hematoxylin/eosin, 20×; scale bar: 50 µm). RSLCs (red arrows) with CD30 negativity (**C**), strong CD20 positivity (**D**), and BCL6 expression (**E**). (immunostaining, 40×; scale bar: 50 µm).

In the setting of FLs, RSLCs may be few or numerous and can be seen between or within the neoplastic follicles [50]. In these cases, the RSLCs have been shown to have identical IGH gene rearrangements as neoplastic centrocytes and centroblasts, suggesting a common cell of origin [51]. Moreover, FL often presents with stromal fibrosis, especially in retroperitoneal or perinephric locations [50] (Supplementary Table S3; Figure 9).

#### 4.1.3. Clue for Differential Diagnosis with cHL

In cHL, RSC is strongly and homogeneously CD30 positive, whereas B cell markers, when present, demonstrate a variable pattern of expression and are commonly weaker than CD30 [5,7]. Conversely, in FL RSLC shows strong and homogeneous expression of B cell markers, whereas CD30 is usually weaker [50]. The concomitant expression of germinal center markers (i.e., CD10 and BCL6) is present in RSLC and virtually never seen in RSC and [5,50]. RSLCs, when associated with FL, may express CD10 and have identical IGH gene rearrangements of the neoplastic germinal center cells. Unlike FL, cHL is characterized by inflammatory infiltrate composed of a mixed population of eosinophils, plasma cells, histiocytes, and small lymphocytes without cytological atypia [7,50] (Supplementary Table S3).

### 4.2. Primary Cutaneous Marginal Zone Lymphoma (PCMZL)

#### 4.2.1. Epidemiology and Clinical Features

PCMZL is a distinct subtype of indolent cutaneous B cell lymphoma, limited to skin, included in the group of extranodal marginal zone lymphoma of mucosa-associated lymphoid tissue (MALT lymphoma) in the current WHO classification, accounting for approximately 30% of cutaneous B cell lymphomas [9]. Patients are most commonly young to middle-aged adults (median age 39–55 years), typically presenting with solitary or grouped red-brown papules and/or plaques with a predilection for the trunk and upper extremities [52]. In endemic areas, an association with *Borrellia burgdorferi* infection has been documented [53]. Therapy is usually local and skin-directed, including surgical excision, radiotherapy, and intralesional steroids. In general, recurrences are common (44–71% of patients) at distant sites from the initial presentation, but the prognosis is excellent [54]. As the disease is indolent, watchful waiting can also be an option in some cases [55] (Supplementary Table S3).

#### 4.2.2. Histological Findings and Immunophenotype

Microscopically, PCMZL presents as a nodular and/or diffuse dermal infiltrate, in some cases extending into the subcutis, with perivascular or periadnexal infiltration pattern [9,52]. The infiltrate is usually polymorphous, composed of small- to medium-sized neoplastic “marginal zone cells” (centrocyte-like cells), with indented nuclei and abundant clear cytoplasm, admixed with few larger neoplastic cells (centroblast-like or plasmablasts) and a variable component of plasmacytoid lymphocytes and plasma cells at the periphery of the infiltrate [52]. Plasma cells may also commonly be observed in the superficial dermis lining up along a grenz zone. Numerous reactive CD3 T lymphocytes are usually present with other inflammatory cells, including histiocytes and eosinophils [54]. The epidermis is typically spared without epidermotropism and lymphoepithelial lesions [52,54]. Reactive lymphoid follicles surrounded by pale-appearing areas of expanded “marginal zone cells” are often evident at low magnification [52]. The plasmacytic variant is characterized by an infiltrate composed almost exclusively of plasma cells. Neoplastic lymphocytes and plasmacytoid lymphocytes are positive for B cell markers, including CD20 and CD79α; BCL2 is generally positive. Germinal center markers such as CD10 and BCL6 are negative as well as CD5. A diagnostic key feature is the monoclonal light chains restriction, which is almost invariably detected and characteristically highlights monotypic cells at the periphery of nodules. In this context, a ratio of 10:1 kappa to lambda (or lambda to kappa) is generally used as a threshold for monoclonality [52]. Monoclonal rearrangement of IGH genes is detected in 50% to 60% of cases [9]. In contrast with other MALT lymphomas, PCMZL has only rarely been found to harbor the t(11;18); however, a subset of cases has been shown to harbor translocations involving IGH and various partners, including t(14;18) involving *BCL2* and t(3;14) involving *IGH* and *FOXP1* [52]. 

Large, scattered, clustered, or diffuse CD30+ cells throughout the tumoral infiltrate with RSLC morphology, and occasionally even with histological features, mimicking cHL have been observed [54]. RSLCs are often positive for CD20, PAX-5 and BCL2 while negative for BCL6 and CD10 and are surrounded by pseudorosettes of CD3+ T lymphocytes [54] (Supplementary Table S3).

#### 4.2.3. Clues for Differential Diagnosis with cHL

The rare cutaneous presentation of cHL is always secondary to systemic disease, while PCMZL is skin-restricted [54]. Moreover, strong positivity for CD20, PAX5, and EBV negativity in RSLCs is unusual in cHL [7,9]. Clinically, the presence of CD30 + RSLCs in PCMZL is associated with a more aggressive behavior, with multiple recurrences and large tumor masses [54] (Supplementary Table S3).

### 4.3. Chronic Lymphocytic Leukemia/Small Lymphocytic Lymphoma

#### 4.3.1. Epidemiology and Clinical Features

CLL/SLL is classically a disease of the elderly (median age 71 years), with a slight predominance in males and in Caucasians. CLL/SLL often initially has an indolent course where a wait-and-see approach is appropriate [56,57]. CLL usually presents as an incidental finding during a routine complete blood count. On the other hand, lymphadenopathy is the first manifestation of SLL [56]. Non-specific B symptoms may help to identify the patient needing treatment [56]. When treatment is appropriate, newer therapies target the B cell receptor pathway as well as cellular regulator proteins [56] (Supplementary Table S3).

#### 4.3.2. Histological Findings and Immunophenotype

The hallmark of SLL is a diffuse proliferation of monomorphic, small, round lymphocytes with scattered larger nucleolated cells (prolymphocytes and paraimmunoblasts), which commonly form aggregates known as proliferation centers or pseudo-follicles [58]. Small lymphocytes are positive for B-markers (CD20, CD79α, CD5, LEF1, CD23, the latter stronger in the proliferation centers); Zap 70 expression has a prognostic value [9]. Of note in CLL, CyclinD1-positive cells can be found in proliferation centers.

The presence of scattered RSLCs within a monomorphous, typical background of CLL/SLL represents an unusual pattern referred to as “CLL/SLL with RSLCs” [59]. In detail, RSCs in CLL/SLL are encountered in two different forms. Type I is defined as a typical SLL background with scattered and sporadic RSLCs, whereas type II shows segregated areas of typical RSCs within a polymorphous inflammatory background, distinct from typical CLL/SLL areas [60]. This latter pattern can be considered as an early event in the transformation of CLL/SLL into high grade lymphoma [47]. Less than 1% of patients with CLL/SLL develop cHL, and it has been suggested that exposure to immunosuppressive chemotherapy may increase the risk of HL transformation [61]. RSLCs or RSCs express CD30, CD15, and PAX5; EBV is often positive; moreover, RSCs may be CD20 positive with variable intensity, in 20–30% of cases [62] (Supplementary Table S3).

#### 4.3.3. Clues for Differential Diagnosis with cHL

Presence of scattered EBV positive RSLCs in the background of SLL, by itself, does not fulfill the criteria for a diagnosis of cHL [9], but RSCs in a typical, polymorphous inflammatory background made of T cells and histiocytes, with or without abundant eosinophils and tumor necrosis is diagnostic for cHL [61] (Supplementary Table S3).

### 4.4. Primary Cutaneous Follicle Center Lymphoma (PCFCL)

#### 4.4.1. Epidemiology and Clinical Features

PCFCL accounts for approximately 50% to 60% of primary cutaneous B cell lymphomas and typically occurs in middle aged to older people [9,63]. Although PCFCL can be seen occasionally in young adults, the disease is extremely rare in children [64]. Patients with PCFCL usually present with erythematous papules, plaques, or tumors in the head, neck, or trunk, but any site can be involved, including legs [9,52]. Lesions can be either solitary or, rarely, multiple; multiple lesions are often grouped [52]. PCFCL is associated with an excellent prognosis [52]. BM involvement is documented in 5% of cases [52].

For solitary lesions, skin-directed therapies are typically used (radiotherapy or surgical excision or intralesional steroid injection and targeted cryotherapy) [65]. In widespread disease, rituximab and chemotherapeutic agents are sometimes used with varying success [65] (Supplementary Table S3).

#### 4.4.2. Histological Findings and Immunophenotype

PCFCL shows variation in both cytomorphologic features and architecture [9,52]. Follicular/nodular, diffuse, or mixed growth patterns are described. Regardless of architectural pattern, the predominant neoplastic cells are medium to large-sized centrocytes (cleaved cells) with angulated, irregular, and sometimes multilobulated nuclei; variable numbers of admixed centroblasts may also be seen, as few immunoblasts with large, central nucleoli [9,52]. All histopathologic variants of PCFCLs are characteristically positive for standard B cell markers as CD20, CD79α, PAX5, and BCL6 with variable positivity for CD10. MUM1/IRF4 and BCL2 are usually negative. CD21-positive dendritic cell networks strongly support the diagnosis of PCFCL [9,52].

Isolated RSLCs are described in PCFCL, but their significance has not been well investigated yet [66,67] (Supplementary Table S3).

#### 4.4.3. Clues for Differential Diagnosis with cHL

First of all, a skin-limited disease is strongly suggestive for PCFCL and very unusual in cHL. When present, the RSLCs are CD45/LCA+ and coexpress CD30, CD20 (very strongly), PAX5, BCL2 CD79α, BCL6, and MUM1/IRF4; CD15 and LMP1 are negative [66] (Supplementary Table S3).

## 5. Rare Lymphoproliferative Diseases

### 5.1. Nodal Involvement by CD30+ Cutaneous Lymphoproliferative Disorders (CD30+ LPDs)

#### 5.1.1. Epidemiology and Clinical Features

CD30+ LPDs include primary cutaneous anaplastic large cell lymphoma (C-ALCL), lymphomatoid papulosis (LyP), and a subset of transformed Mycosis Fungoides (MF) [9,68]. MF is a cutaneous-limited T cell lymphoma, typically presenting with flat, scaly lesions or patches [9]. Patients with disseminated plaques, tumors, or both may develop visceral disease, with lymph node involvement, hepatosplenomegaly, or infiltrates in other organs [68]. A minority of patients with MF develop the Sézary syndrome, with diffuse erythroderma, diffuse lymphadenopathy, and leukemic involvement [9,68,69]. LyP lesions appear as small self-healing papules, with a necrotic center, often in clusters and recurring in the same region of the body [9,68]. Patients with C-ALCL generally present with solitary or localized ulcerating tumors or nodules [9].

In CD30+ LPDs, dissemination to regional lymph nodes can develop in up to 10% of patients [69] with no differences in prognosis [70]. Advanced-stage MF including systemic lymphadenopathy, however, has a poorer prognosis, and large cell transformation is associated with worse outcome [69]. Nodal involvement by cutaneous CD30+ LPDs must be distinguished from nodal involvement by cHL, which differs in clinical behavior, prognosis, and therapeutic approach [71].

#### 5.1.2. Histological Findings and Immunophenotype

When excisional lymph node biopsy is performed in diffuse CD30+ LPDs, involved lymph nodes may demonstrate effacement of the nodal architecture, causing a nodular appearance or fibrosis, such as in cHL [68]. Necrosis and adherence of the excised node to the surrounding fat, denoting extracapsular extension, can be seen [70]. The classic form of RSLC and its variants can be identified [68]. Consequently, cHL represents a pitfall in the diagnosis of lymph node involvement by CD30+ LPDs, and vice versa. A correct diagnosis has a direct impact on the patient’s management [68]. MF cells are typically CD4+ and CD8-; they also express most T cell antigens including CD2, CD3, CD5, CD43, and T cell receptor αβ, but they are often negative for CD7. Large cells of transformed MF express CD30 in 48% to 70% of cases [70]. C-ALCL and large cell forms of LyP are CD30+, often CD4+, with variable expression of other T cell antigens. Loss of T cell antigen expression and aberrant expression of CD15 and PAX5 is noted in a subset of cases [72].

#### 5.1.3. Clues for Differential Diagnosis with cHL

Clinically, the presence of B symptoms and extensive nodal disease, particularly with mediastinal involvement, favors the diagnosis of cHL. A previous history of MF, LyP, and C-ALCL favors a nodal involvement by CD30+ LPDs [70,71,73]. Complete and deep anamnesis is the most useful criteria.

Some cases, however, are difficult to diagnose for the presence, in the lymph node, of thick fibrous bands, characteristic of cHL, but these bands are described also in the nodal involvement by CD30+ LPDs [74]. Anaplastic cells in CD30+ LPDs vary in size and may have multiple nucleoli, whereas RSCs and their variants are more uniformly large and have 1 or 2 large eosinophilic nucleoli [68]. Moreover, RSLCs in CD30+ LPDs may be set in a polymorphic inflammatory background with eosinophils, neutrophils, plasma cells, and histiocytes similar to mixed cellularity or nodular sclerosis-type cHL and, in addition, can co-express CD30, CD15, and PAX5 [68,72], whereas cHL may have aberrant T antigen expression and is CD15 negative in a subset of cases [73]. However, PAX5 is expressed in 90% to 95% of cHLs [5,9], while aberrant expression of PAX5 in CTCL, also if reported, is rare [68]. LMP1 is negative in transformed MF, LyP, and ALCL-C but often positive in cHL [68]. If present, sinusoidal involvement by the large cells favors CD30+ LPDs [74]. CD43 is rarely aberrantly expressed in cHL (<5%) but is one of the most sensitive markers for CD30+ LPDs [5,9]. Finally, RSCs in cHL commonly lack CD45/LCA, unlike RSLCs in CD30+ LPDs [68].

### 5.2. Cutaneous Localization of AITL

#### 5.2.1. Epidemiology and Clinical Features

Cutaneous involvement is seen in approximately 50% of patients with AITL and is usually secondary to a systemic disease. Patients with cutaneous involvement classically present a transient morbilliform eruption or other cutaneous manifestations such as papules, nodules, urticarial plaques, and erythroderma [75]. EBV infected B cell expansion is typically observed in AITL [76].

#### 5.2.2. Histological Findings and Immunophenotype

Cutaneous localization of AITL shows various histological pictures. Non-specific patterns include perivascular eosinophilic infiltrate or leukocytoclastic vasculitis [77]. Prominent granulomatous reaction may also be observed, sometimes mimicking infectious lesions [78]. Other patterns are suggestive of cutaneous lymphoma and may present as sparse superficial perivascular or dense pleomorphic infiltrate with atypical lymphocytes [75,76]. Due to the scarcity of neoplastic T cells, establishing a diagnosis of AITL may be challenging in cutaneous lesions [76].

Skin biopsies show dermal infiltrate of CD20− and EBER-positive medium- to large-sized atypical lymphoid cells hiding neoplastic T cells [76]. The RSLCs are positive for CD30, CD15, and EBV, and have a partially preserved B cell program (focal and weak PAX5 positivity, CD20 negativity) as described in cHL [76]. Although skin occurrence of cHL is rare, it has been described in advanced stages with the same clinical skin damages as cutaneous AITL [79]. RSCs harbor the classical morphology and immunophenotype; nevertheless, because of its rarity and diagnostic challenge in skin localization, a primary diagnosis of cHL on skin biopsy should never be suggested without a systemic diagnosis on lymph node [76].

#### 5.2.3. Clues for Differential Diagnosis with cHL

Clinical history is the main key for correct differential diagnosis [75,76,79].

### 5.3. Composite Lymphoma (CL)

CL is defined as the coexistence of two or more morphologically and immunophenotipically distinct lymphomas in a single anatomic organ, and it is uncommon, especially in extranodal sites [80]. CL, accounting for approximately 1% to 4% of lymphomas, consists of a combination of HL with NHL or two different NHLs [81]. NHL combined with cHL is much more uncommon than other combined NHLs [82]; in this setting, cHL is more frequently combined with B cell lymphomas [83], in particular FL, CLL/SLL and DLBCL [81,84,85].

Another CL type is PMBL combined with cHL [86]. The existence of this form as well as GZL suggests lineage plasticity between the two molecularly related entities, cHL and PMBL [87], driven by abnormalities involving the JAK/STAT [88] and/or other molecular pathways [89].

Coexistence of DLBCL and cHL in the same anatomic location has been reported occasionally, often showing EBV positivity, suggesting an origin from a commonly EBV-infected progenitor cell [85,90]. Molecular studies have proved that they may or may not be clonally related [91,92].

MCL combined with cHL ha been described in both classical [93] and blastoid variant [46]. While earlier reports hypothesized that RSCs arise as a direct descendent of the MCL clone, new studies support progression from a common clonal progenitor cell with subsequent distinct transforming events [94].

The least frequent CL is T cell NHL combined with cHL [95], specifically, Peripheral T cell Lymphoma (PTCL) and cHL [96]. Due to rarity, this entity has not been well investigated yet [97].

### 5.4. EBV-Positive Mucocutaneus Ulcer (EBV+ MCU)

#### 5.4.1. Epidemiology and Clinical Features

EBV+ MCU has been included as a provisional entity in the current WHO classification among the EBV lymphoproliferative disorders [9]. EBV+ MCU is associated with immunosuppression. All patients described in the original series were receiving immunosuppressive medication or had age-related immunosenescence, with a median age of 77 years [98]. Patients presented well-circumscribed, often painful, ulcerating lesions arising at mucosal or cutaneous sites. Oropharyngeal mucosa is the most frequent site of presentation [98]. Cutaneous involvement is often perioral, but acral sites, the trunk, and gastrointestinal tract may also be affected [99]. Importantly, and irrespective of site, EBV+ MCU is typically a superficial lesion and no mass lesion is detectable on clinical examination or imaging. The presence of lymphadenopathy and/or spleen, liver or bone marrow involvement should also lead to a presumptive diagnosis of EBV+ MCU being questioned [99]. EBV+ MCU generally follows an indolent course with spontaneous regression or remission upon reduction of immunosuppressive drugs [99,100].

#### 5.4.2. Histological Findings and Immunophenotype

EBV+ MCU typically features a polymorphous infiltrate including large atypical cells with Hodgkin-like appearance [9,98]. These atypical cells are admixed with small lymphocytes, plasma cells, histiocytes, neutrophils, and eosinophils. Plasmacytoid apoptotic cells and necrosis are present in a significant proportion of cases [9,99]. Reactive squamous epithelial atypia and pseudoepitheliomatous hyperplasia is often present [9,99]. The deep margin of the ulcer is usually well defined by a rim of CD8 positive T lymphocytes. The RSLCs are generally strongly EBV and CD30 positive with variable expression of CD15 and B cell markers such as CD20. The cells usually express PAX5, MUM1, OCT2, with variable BOB1 expression. EBV typically reveals a type II or type III latency pattern [101]. In situ hybridization for EBV (EBER) shows intense and diffuse positive staining [98,101]. About one-third of cases show clonal Ig rearrangement, one-third clonal T cell rearrangement, and one-third a restricted T cell pattern [98,99].

#### 5.4.3. Clues for differential diagnosis with cHL

EBV+MCU may resemble cHL, morphologically and phenotypically, with the RSLCs expressing CD30, PAX5, and EBV. However, the clinical criteria together with the circumscribed and superficial nature of the ulcer, without a tumor-forming lesion are features in favor of EBV+ MCU. Another useful clue is the sharp deep margin of the ulcer rimmed by small T lymphocytes. Unlike EBV+ MCU, cHL is always a tumor-forming lesion. Additionally, the extreme rarity of cHL presenting as extranodal disease in the absence of nodal involvement must be considered [9,100].

## 6. Conclusions

cHL and NHL have different epidemiologic, clinical, therapeutic, and prognostic findings. RSC in appropriate milieu is a pathognomonic feature of cHL, but RSLCs with similar morphologic and immunophenotipic profile can also been found in NHLs. A correct morphological and immunohistochemical approach, integrated with clinical and serological data, can help to distinguish cHL from NHLs leading to the appropriate management of patients.

## Supplementary Materials

The following are available online at https://www.mdpi.com/2075-4418/10/12/1019/s1, Table S1: Clinical, morphologic and immunohistochemical comparation between cHL and T cell lymphomas; Table S2: Clinical, morphologic and immunohistochemical comparation between cHL and High-Grade B cell Lymphomas; Table S3: Clinical, morphologic, and immunohistochemical comparation between cHL and Low-Grade B cell Lymphomas.

## Abbreviations

ABVD	Doxorubicin, Bleomycin, Vinblastine, and Dacarbazine
AITL	Angioimmunoblastic T cell Lymphoma
ALCT	Anaplastic Large T cell Lymphoma
ALK+ ALCL	Anaplastic Lymphoma Kinase-positive Anaplastic Large Cell Lymphoma
ALK− ALCL	Anaplastic Lymphoma Kinase-negative Anaplastic Large Cell Lymphoma
ALK+ LBCL	Anaplastic Lymphoma Kinase-positive Large B cell Lymphoma
BIA-ALCL	Breast Implant-Associated Anaplastic Large Cell Lymphoma
BM	Bone Marrow
C-ALCL	Primary Cutaneous Anaplastic Large Cell Lymphoma
CD	Castleman Disease
CD30+ LPDs	CD30+ Cutaneous Lymphoproliferative Disorders
cHL	classical Hodgkin Lymphoma
CHOP	Cyclophosphamide, Doxorubicin, Vincristine, and Prednisone
CL	Composite Lymphoma
CLL/SLL	Chronic Lymphocytes Leukemia/Small lymphocytes lymphoma
DLBCL, NOS	Diffuse Large B cell Lymphoma, Not Otherwise Specified
EBER	EBV-encoded small RNA
EBV	Epstein–Barr Virus
EBV+ DLBCL	EBV-positive Diffuse Large B cell Lymphoma, not otherwise specified
EBV+ MCU	EBV-positive mucocutaneus ulcer
FDC	follicular dendritic cell
FL	Follicular Lymphoma
F-PTCL	Follicular Peripheral T cell Lymphoma
GZL	Gray Zone Lymphoma
HHV8	Human Herpes Virus 8
IGH	Immunoglobulin Heavy Chain
LBCL	Large B cell Lymphoma
LDH	Serum Lactase Dehydrogenase
LG	Lymphomatoid Granulomatosis
LyP	Lymphomatoid Papulosis
LMP1	Latent Membrane Protein-1
MALT	Mucosa-associated lymphoid tissue Lymphoma
MCL	Mantle Cell Lymphoma
MF	Mycosis Fungoides
NLPHL	Nodular Lymphocytes Predominant Hodgkin Lymphoma
NHL	Non-Hodgkin lymphoma
OS	Overall Survival
PCMZL	Primary Cutaneous Marginal Zone Lymphoma
PCFCL	Primary Cutaneous Follicle Center Lymphoma
PEL	Primary Effusion Lymphoma
PMBL	Primary Mediastinal (Thymic) Large B cell Lymphoma
PTCL	Peripheral T cell lymphoma
RSC	Reed–Sternberg Cell
RSLC	Reed–Sternberg-like cells
TCR	T cell Receptor
TFH	T-follicular helper derived
THRLBCL	T cell/Histiocyte-Rich Large B cell Lymphoma
WHO	World Health Organization
