# Supplementary material for: Hodgkin Reed–Sternberg-Like Cells in Non-Hodgkin Lymphoma
Source: Diagnostics (Basel). 2020 Nov 27;10(12):1019. doi: 10.3390/diagnostics10121019 (PMC7760963; doi:10.3390/diagnostics10121019)
Supplement: Supplementary file 1 [file diagnostics-10-01019-s001.pdf]

Table S1a. Clinical comparation between cHL and T-cell lymphomas.

	Age	Symptoms	Localization	BM Involvement	Prognosis	Treatment
**cHL**	Younger Middle age	B-Symptoms	Typical: lymph nodes Other: mediastinum	Unusual	Good	ABVD; RT
**ALK+ALCL**	Children Young adults	Severe systemic symptoms	Lymph nodes; skin; bone; soft tissues	Usual	Good	CHOP-like
**ALK-ALCL**	Adults	B-Symptoms	Lymph nodes; skin; liver; lung	Unusual	Often relaps	CHOP
**BIA-ALCL**	8-10 years after breast implant	Local mass/seroma	Breast	Unusual	Indolent	Surgical excision
**AITL**	Elderly	Ascites; skin rush; autoimmune-like manifestations	Lymph nodes Visceral organs	Usual	Poor	Not established
**F-PTCL**	None	As AITL	As AITL; inguinal lymph nodes	Usual	Poor	Not establisched

Table S1b. Morphologic comparation between cHL and T-cell lymphomas.

	Growth Pattern	Neoplastic Component	Background	Morphological Hallmark	Genetic Hallmark
**cHL**	Frequently nodular	RSC	Lymphocytes, histiocytes,granulocytes	Isolate RSCs in appropriate background	none
**ALK+ALCL**	Common pattern: diffuse/infiltrative In Hodgkin-like pattern: nodular	Large T-cells CD30+ ALK+	Common pattern: isolate lymphocytes Hodgkin-like pattern: polymorphous infiltrate	Clusters of large CD30+/ ALK+ cells; horse-shoe shaped nuclei	ALK gene rearrangement
**ALK-ALCL**	Diffuse/infiltrative	Large T-cells CD30+ ALK-	Sclerosis and eosinophilia	As ALK+ ALCL	none
**BIA-ALCL**	Diffuse	Medium-to large-sized T-cells	Fibrinoid material and necrosis; inflammatory infiltrate	Non-cohesive sheets of malignant cells	none
**AITL**	Diffuse with capsular/perinodal infiltration	Small-to medium-sized T cells clustering close to vessels	Histiocytes, RSLCs (immunoblasts), eosinophils, plasma cells	Marked proliferation of arborizing high endothelial venules	none
**F-PTCL**	Follicular-like progressive transformation of germinal center	Small-to medium-sized T-cells	None	Follicles involved	none

Table S1c. Immunohistochemical comparation between cHL and T-cell lymphomas.

	RSC in cHL	RSLC in ALK+ALCL	RSLC in ALK-ALCL	RSLC in BIA-ALCL	RSLC in AITL	RSLC in F-PTCL
**B-markers**	Negative. CD20 weak <20%	Negative	Negative	Negative	CD20+; CD79alfa+	CD20+ (32%)
**T-markers**	Negative	CD2+, CD4+, CD5+	Negative GranzymeB+, CD43+	Incomplete T-cell phenotype/CD4+/NK	Negative	Negative
**CD30**	Positive	Positive	Positive	Positive	Positive	Positive
**CD15**	Positive	Rarely positive	Negative	Occasionally positve	Positive	Rarely positive
**LMP1**	Positive	Negative	Negative	Negative	Occasionally positive	Positive (47%)
**PAX5**	Positive (weak)	Negative	Negative	Occasionally positive	Positive	Positive (strong)
**CD45**	Negative	Variably positive	Positive	Positive	Positive	Positive
**MUM1**	Positive	Negative	Negative	Negative	Occasionally positive	Positive
**EMA**	Negative	Positive	Rarely positive	Positive (43-90%)	Negative	Negative
**Hallmark**	CD30+/CD15+	ALK+	Perforin+; CD56+; Clusterin+	TCR genes rearrangements	Background of T-cell CD10+, BCL6+; TRC gene rearrangements	Follicular proliferation of small lymphocytes expressing T-markers

Table S2a. Clinical comparation between cHL and High Grade B-cells Lymphomas.

	Age	Symptoms	Localization	BM Involvement	Prognosis	Treatment
**cHL**	Younger Middle age	B-Symptoms	Typical: lymph nodes Other: mediastinum	Unusual	Good	ABVD; RT
**DLBCL, NOS**	Elderly	B-Symptoms	Typical: lymph nodes Other: Gastro-Intestinal tract	10-20% Concordant (DLBCL) Discordant (Low Grade)	Variable (see in the text)	R-CHOP; autologous stem cell transplantation
**THRLBCL**	Middle	B-Symptoms; Hepatosplenomegaly	Lymph nodes	Common	Poor	R-CHOP
**ALK+LBCL**	All age	B-Symptoms	Typical: lymph nodes Other: Mediastinum	25-30%	Poor	CHOP
**PMBL**	35-37 y.o.	Mediastinic syndrome	Mediastinum Lymph nodes	Uncommon	Variable (see in the text)	Not established
**GZL**	<30 y.o.	Mediastinic syndrome	Mediastinum Lymph nodes	Uncommon	Poor	CHOP
**LG**	46-48 y.o. immunocompromised	Fever; cough	Lungs	Uncommon	Variable (see in the text)	Variable (see in the text)
**PEL**	40-45 y.o. Immunocompromised	Cavitary form: effusion; extracavitary form: mass related	Cavitary: pleural, pericardual, peritoneal.	Uncommon	Poor	Not established
**EBV+DLBCL**	Elderly	Organ-related	Visceral organs	Common	Poor	As DLBCL, NOS
**MCL**	66 y.o.	B-Symptoms	Lymph nodes Extranodal organs	Common	Poor	R-HyperCVAD R-MA

Table S2b. Morphologic comparation between cHL and High Grade B-cells Lymphomas.

	Growth Pattern	Neoplastic Component	Background	Morphological Hallmark	Genetic Hallmark
**cHL**	Nodular	RSC	Lymphocytes, histiocytes, granulocytes	Isolate RSCs in appropriate background	None
**DLBCL, NOS**	Diffuse	Large-to medium-sized B-cells	Small T-cells and histiocytes	Large-to medium-sized B cells effacing lymph node	None
**THRLBCL**	Diffuse	RSLC with B immunophenotype	Host T lymphocytes with/without epithelioid histiocytes	Host T lymphocytes with/ without epithelioid histiocytes	None
**ALK+LBCL**	Diffuse or sinusoidal	Monomorphic B-cells ALK+	None	Plasmablastic like cells effacing lymph node	ALK gene rearrangement
**PMBL**	Diffuse	Medium-to large B-cells CD23+	Variable degree of sclerosis and collagen band.	Variable degree of sclerosis and collagen band.	None
**GZL**	Wide variation	B-cells with morphologic and immunophenotypic findings intermediate between cHL and DLBCL	Paucity of inflammatory cells	High tumor cell content	None
**LG**	Angiocentric and angiodistructive	EBV+ B cells	Occasional plasma cells; necrosis	Angiocentric and angiodistructive infiltrate	None
**PEL**	Isolate cells	HHV8+ large-sized B cells	None	HHV8+ large-sized B cells	None
**EBV+DLBCL**	Diffuse	EBV+ large-sized B cells	Numerous small lymphocytes and histiocytes and plasma cells	EBV+ large-size B cells	None
**MCL**	Nodular or diffuse	Cyclin D1+ small-to medium-sized B cells	None	Cyclin D1+ small-to medium size B cells	t(11-14)

Table S2c. Immunohistochemical comparation between cHL and High Grade B-cells Lymphomas.

	RSC in cHL	RSLC in DLBCL, NOS	RSLC in THRLBCL	RSLC in ALK+LBCL	RSLC in PMBL	RSLC in GZL	RSLC in LG	RSLC in PEL	RSLC in EBV+DLBCL	RSLC in MCL
**B-markers**	Negative. CD20 weak <20%	Positive, strong	Positive, strong	Negative	Positive, strong	Variable	Positive	Positive in extracavitary	Positive, strong	Positive
**T-markers**	Negative	Negative	Negative	Negative	Negative	Negative	Negative	Aberrant	Negative	CD5+
**CD30**	Positive	Positive (10-15%)	Usually negative	Usually negative	Usually positive, weak	Variable	Usually positive	Usually positive	Positive (30%)	Usually positive
**CD15**	Positive	Negative	Usually negative	Negative	Usually negative	Variable	Negative	Negative	Rarely positive	Variable
**LMP1**	Positive	Negative	Negative	Negative	Negative	Variable	Positive	Negative	Positive	Variable
**PAX5**	Positive, weak	Positive, strong	Positive	Negative	Positive, strong	Variable	Positive	Positive	Positive	Positive
**CD45**	Negative	Positive	Positive	Negative	Positive	Variable	Positive	Positive	Positive	Positive
**MUM1**	Positive	Negative	Negative	Positive	Positive	Variable	Variable	Positive	Positive	Negative
**EMA**	Negative	Negative	Positive (30%)	Positive (90%)	Negative	Variable	Negative	Usually positive	Usually negative	Negative
**Hallmark**	CD30+/CD15+ (BOB1- OCT2-)	BOB1+ and OCT2+	BCL6+	ALK+; BOB1+ and OCT2+	CD23+; BOB1+ OCT2+	None	None	HHV8+; BOB1+ and OCT2+	EBER+	CD5+; cyclinD1+ SOX11+

Table S3a. Clinical comparation between cHL and Low Grade B-cells Lymphomas.

	Age	Symptoms	Localization	BM Involvement	Prognosis	Treatment
**cHL**	Younger Middle age	B-Symptoms	Typical: lymph nodes Other: mediastinum	Unusual	Good	ABVD; RT
**FL**	60-65 y.o.	B-Symptoms	Cervical and Abdominal Lymph nodes	Usual	Good	Depending on stage (see in the text)
**PCMZL**	Young-to middle age	Solitary or grouped skin lesions	Skin	Very unusual	Good	Local skin-directed
**CLL/SLL**	Elderly	Lymphadenopathy	Lymph nodes	Usual	Good	Wait-and-see
**pcFCL**	Middle age to older	Skin lesions	Skin	5%	Good	Local skin-directed

Table S3b. Morphologic comparation between cHL and Low Grade B-cells Lymphomas.

	Growth Pattern	Neoplastic Component	Background	Morphological Hallmark	Genetic Hallmark
**cHL**	Nodular	RSC	Lymphocytes, histiocytes, granulocytes	Isolate RSCs in appropriate background	none
**FL**	Nodular; Diffuse; Mixed	Small-to medium-sized B-lymhocytes of Germinal Center	Reactive T-cells, FDCs and histiocytes	Nodular/Diffuse proliferation of small-to medium-sized lymphocytes	t(11;14)
**PCMZL**	Nodular/diffuse dermal infiltration	Small-to medium-sized 'marginal zone cells' and larger B-cells	Plasmacytoid lymphocytes, plasma cells, T-lymphocytes, histiocytes	Grenz zone without epidermotropism	None
**CLL/SLL**	Nodular	Monomorphic small B cells with scattered larger cells	Monomorphous lymphocytes proliferation	Proliferation centers	None
**pcFCL**	Nodular, diffuse, mixed	Medium-to large-sized centrocytes and immunoblasts	In follicular pattern: reactive small lymphocytes	Skin-restricted lesions	None

Table S3c. Immunohistochemical comparation between cHL and Low Grade B-cells Lymphomas.

	RSC	RSLC in FL	RSLC in PCMZL	RSLC in CLL/SLL (Type I)	RSLC in pcFCL
**B-markers**	Negative.<br>CD20 weak <20%	Positive; CD10+, BCL6+, BCL2+	CD20+ strong and BCL2+	CD20 in 20-30%	CD20 strong
**T-markers**	Negative	Negative	Negative	Negative	Negative
**CD30**	Positive	Focal and weak	Positive	Positive	Positive
**CD15**	Positive	Negative	Negative	Positive	Negative
**LMP1**	Positive	Negative	Negative	Usually positive	Negative
**PAX5**	Positive, weak	Positive	Positive	Positive	Positive
**CD45**	Negative	Positive	Positive	Positive	Positive
**MUM1**	Positive	Negative	Negative	Negative	Positive
**EMA**	Negative	Negative	Negative	Negative	Negative
**Hallmark**	CD30+/CD15+	CD10+, BCL6+, BCL2+	Strong expression of B-markers	None	Strong expression of B-markers
